# Biological activity reduction and mitochondrial and lysosomal dysfunction of mesenchymal stem cells aging in vitro

**DOI:** 10.1186/s13287-022-03107-4

**Published:** 2022-08-13

**Authors:** Ge Zhang, Yuli Wang, Jianhua Lin, Bo Wang, Ali Mohsin, Zhimin Cheng, Weijie Hao, Wei-Qiang Gao, Huiming Xu, Meijin Guo

**Affiliations:** 1 State Key Laboratory of Bioreactor Engineering, East China University of Science and Technology, P.O. Box 329#, 130 Meilong Road, Shanghai, 200237 People’s Republic of China; 2 State Key Laboratory of Oncogenes and Related Genes, Renji-MedX Clinical Stem Cell Research Center, Ren Ji Hospital, School of Medicine, Shanghai Jiao Tong University, 160 Pujian Road, Shanghai, 200127 China; 3 Department of Vascular Surgery, Ren Ji Hospital, School of Medicine, Shanghai Jiao Tong University, Shanghai, 200127 China; 4 Department of Obstetrics and Gynecology, Ren Ji Hospital, School of Medicine, Shanghai Jiao Tong University, Shanghai, 200127 China; 5 Med-X Research Institute and School of Biomedical Engineering, Shanghai Jiao Tong University, Shanghai, 200030 China

**Keywords:** Human umbilical cord mesenchymal stem cells, Cell activity, Mitochondrial dysfunction, Lysosomal dysfunction

## Abstract

**Background:**

Mesenchymal stem cells (MSCs) have been extensively used for the treatment of various diseases in preclinical and clinical trials. In vitro propagation is needed to attain enough cells for clinical use. However, cell aging and viability reduction caused by long-time culture have not been thoroughly investigated, especially for the function of mitochondria and lysosomes. Therefore, this study was designed to detect mitochondrial and lysosomal activity, morphological and functional changes in human umbilical cord MSCs (UMSCs) after long-time culture.

**Methods:**

First, we examined cell activities, including proliferation and immigration ability, differentiation potential, and immunosuppressive capacity of UMSCs at an early and late passages as P4 (named UMSC-P4) and P9 (named UMSC-P9), respectively. Then, we compared the mitochondrial morphology of UMSC-P4 and UMSC-P9 using the electronic microscope and MitoTracker Red dyes. Furthermore, we investigated mitochondrial function, including mitochondrial membrane potential, antioxidative ability, apoptosis, and ferroptosis detected by respective probe. Cell energy metabolism was tested by mass spectrometry. In addition, we compared the lysosomal morphology of UMSC-P4 and UMSC-P9 by electronic microscope and lysoTracker Red dyes. Finally, the transcriptome sequence was performed to analyze the total gene expression of these cells.

**Results:**

It was found that UMSC-P9 exhibited a reduced biological activity and showed an impaired mitochondrial morphology with disordered structure,  reduced mitochondrial crista, and mitochondrial fragments. They also displayed decreased mitochondrial membrane potential, antioxidative ability, tricarboxylic acid cycle activity and energy production. At the same time, apoptosis and ferroptosis were increased. In addition, UMSC-P9, relative to UMSC-P4, showed undegraded materials in their lysosomes, the enhancement in lysosomal membrane permeability, the reduction in autophagy and phagocytosis. Moreover, transcriptome sequence analysis also revealed a reduction of cell function, metabolism, mitochondrial biogenesis, DNA replication and repair, and an increase of gene expression related to cell senescence, cancer, diseases, and infection in UMSC-P9.

**Conclusion:**

This study indicates that in vitro long-time culturing of MSCs can cause mitochondrial and lysosomal dysfunction, probably contributing to the decline of cell activity and cell aging. Therefore, the morphology and function of mitochondria and lysosomes can be regarded as two important parameters to monitor cell viability, and they can also serve as two important indicators for optimizing in vitro culture conditions.

**Supplementary Information:**

The online version contains supplementary material available at 10.1186/s13287-022-03107-4.

## Background

Nowadays, mesenchymal stem cells (MSCs) have drawn increasing attention as a promising therapeutic option for various diseases, including immune diseases and non-immune diseases in preclinical and clinical trials [1]. To get an adequate number of cells for transplantation, in vitro culture is required prior to clinical use. However, long-time in vitro culture and repeated propagation usually cause cell aging and affect cell biological activities and therapeutical effects. Previous studies have shown that MSCs exhibit senescence features with enlarged and flattened cell morphology at late passages. The cells in aging show low proliferation ability, a reduction of differentiation potential, immunoregulation capacity, paracrine function, and increased expression of genes and miRNAs related to cell aging, cancers, and diseases [2–4]. In addition, Crisostomo et al. reported that MSCs at early passages (P3) could attenuate rats' myocardial ischemia/reperfusion (I/R) injury. Conversely, MSCs at late passages (P10) aggravate myocardial I/R injury [5]. These studies suggest that MSCs at late passages, showing senescent morphology, might lose therapeutical effects on diseases. Therefore, it is essential to thoroughly assess the biological activity and function of MSCs before clinical application.

Currently, increasing evidence has indicated that mitochondrial dysfunction is associated with aging and many diseases, such as type 2 diabetes, obesity, neurodegeneration, myocardial I/R injury, immune and inflammation disorders, cancers, etc. [6–8]. The mitochondrion is an essential organelle for energy metabolism, calcium homoeostasis, β-oxidation of fatty acid, steroid hormone biosynthesis, generation and scavenging of reactive oxygen species (ROS). In addition, mitochondria play an important role in modulating apoptosis, ferroptosis, and inflammasome activation [9–13]. Mitochondrial dynamics, including mitochondrial fission, fusion, biogenesis, mitophagy (also known as mitochondrial degradation) and transport, coordinately controls the mitochondrial morphology, quantity, quality, and inheritance [14]. Disturbed mitochondrial dynamics affects mitochondrial function, including energy production, cell metabolism, cellular function, and response to external stimulus and stress, closely related to diseases and aging [14, 15]. It is speculated that the long-term culture of MSCs could affect mitochondrial morphology and function.

In addition to mitochondria, the lysosome is also an importing organelle that degrades various extracellular and cellular components by fusing endosome or autophagosome. Among them, lysosomes degrade and recycle intracellular components including nucleic acids, unfolded protein, cytosol portions, and damaged organelles. This process is termed as autophagy [16]. Autophagy plays a crucial role in adapting to metabolism stress, removing damaged proteins and organelles, intracellular pathogens, preventing DNA mutation [17], and scavenging excessive ROS by autolysosome [18]. Increasing evidence indicates that autophagy is required to maintain stemness, repair, remodeling, and metabolism reprogramming of adult stem cells [19, 20]. Recently, lysosome has also been regarded as a signaling regulatory hub, which senses, adapts and responds to changes in substrate metabolism. Therefore, lysosomes play an important role in nutrient sensing, immune cell signal transduction, metabolism, membrane repair, cellular homeostasis, and cell death [21–23]. Lysosomal dysfunction is associated with many aging-related pathologies, including lysosomal storage diseases, neurodegenerative diseases, aging, cancer, and infectious diseases [24–26]. In summary, both mitochondria and lysosomes play important roles in maintaining cellular homeostasis, metabolism balance, and cell functions. Therefore, this study focuses on investigating the morphological and functional changes of mitochondria and lysosomes in human umbilical cord MSCs (UMSCs) after long-time culture, and these changes might be directly related to the decline of cell biological activity and therapeutical effect.

## Materials and methods

### Cell culture

UMSCs are isolated from human umbilical cord tissue from women undergoing caesarean, according to previously described protocols [4]. The women gave informed consent for umbilical cord collection. The collection and subsequent use of umbilical cords was approved by the Human Ethics Committee of Renji hospital, School of Medicine, Shanghai Jiaotong University.

### Cell proliferation assays of UMSCs

UMSCs were seeded into a 96-well plate (2500 cells per well), and the cell proliferation was tested by CCK-8 jit (Dojindo) from day 1 to day 4.

### Identification of the surface markers of UMSCs by flow cytometry

UMSCs were digested and washed twice with PBS and then were stained with isotype control IgG or monoclonal antibodies conjugated to secondary antibodies. The following is the information of antibodies: CD105-APC, CD90-FITC, CD73-FITC, CD34-FITC, HLA-DR-FITC, CD29-FITC, CD45-FITC, CD44-FITC (all from eBioscience). The flow cytometry was performed by using a BD Accuri™ C6 Flow Cytometer (BD).

### β-galactosidase staining

UMSCs were seeded in 12-well plates and cultured up to 80–90% confluence, and SA-β-galactosidase (β-gal) staining was performed with senescence-associated β-gal assay kit (Beyotime) according to the manufacturer’s instructions.

### Cell cycle and apoptosis assays

For cell cycle analysis, the cells were collected, fixed with 70% ethanol overnight at 4 °C, and stained with cell cycle and apoptosis Kit (YEASEN, China). For cell apoptosis assays, the cells were stained using an Annexin V-FITC/PI apoptosis detection Kit (YEASEN, China). Then, the cells were analyzed by Flow Cytometer using a BD Accuri™ C6 Flow Cytometer.

### Colony-formation experiment

UMSCs were seeded into a 6-well plate (200 cells per well) and cultured in a 37 °C incubator for 8–10 days. The cells formed an obvious colony and then were stained with crystal violet Staining solution (Beyotime, China).

### Cell migration analysis

The cell migration experiment was performed in transwell chambers with 8 µm pore size (Corning). UMSCs (1 × 10^4^) were seeded into the upper chamber of transwell in serum-free or UltroGRO-free α-MEM medium (Gibico), and α-MEM medium supplemented with 5% UltroGRO (Helios Bioscience) was placed in the lower chamber. The cells were cultured at 37 °C for 20–24 h and then fixed and stained with crystal violet staining solution.

### Analysis of adipogenic and osteogenic differentiation

To assess the differentiation potential of UMSCs toward osteoblasts and adipocytes, the cells at 80–90% confluence were cultured with osteogenic or adipogenic differentiation medium (STEMCELL Technologies). Osteoblastic and adipocyte differentiation abilities were identified with alizarin red staining and Oil Red O solution, respectively.

### T cell proliferation assay

First, the peripheral blood mononuclear cells (PBMCs) were isolated from fresh peripheral blood with a Ficoll Hypaque density gradient reagent (MD Pacific Biotechnology). The peripheral blood was collected from healthy volunteers who signed informed consent. Second, the PBMCs were labeled with 5 µM carboxyfluorescein succinimidyl ester (CFSE) (Thermo Fisher) and seeded in 24-well plate (5 × 10^5^/well) pre-coated with CD3 and CD28 antibodies (2 µg/mL, Biolegend) in the RPMI 1640 medium supplemented with 10% FBS (Gibico) and 1 ng/mL IL-2 (Perprotech) for 24 h. Next, the PBMCs were co-cultured with UMSCs (1 × 10^5^/well) for 72 h. Finally, PBMCs were collected and stained with CD3-PerCP, CD4-APC, CD8-PE (all from BD Bioscience), then performed flow cytometry. CD4 and CD8 T cell proliferation were analyzed by the frequency of CSFE dilution.

### Mitochondrial and lysosomal staining

Mitochondria and lysosomes were stained with the MitoTracker™ Red CMXRos kit and LysoTracker™ Red DND-99 kit (Thermo Fisher SCIENTIFIC), respectively, according to the manufacturer’s instruction.

### Mitochondrial membrane potential test

Mitochondrial membrane potential was measured with tetramethylrhodamine, methyl ester (TMRM), a fluorescent, cell-permeant cationic dye (Beyotime) according to the manufacturer’s instruction. The cells were stained with TMRM and analyzed by flow cytometry.

### Reactive oxygen species (ROS) and lipid ROS test

Total ROS levels were measured with fluorescent probe 2′,7′-dichlorodihydrofluorescein diacetate (H2-DCFDA, Life Technology). The lipid peroxidation levels were tested by BODIPY ® Lipid Probes (All from Thermo Fisher SCIENTIFIC).

#### GSH assay

The cells of UMSCs were harvested for GSH analysis using a total GSH/GSSH assay kit (Beyotime) according to the manufacturer’s instruction.

### Analysis of lysosomal endocytosis

To test lysosomal endocytosis, UMSCs were cultured in basic medium with FITC-conjugated beta-amyloid peptide (1–42) (Aβ-FITC, PLLABS) for 4 h and then washed with PBS. After that, UMSCs were observed by an inverted fluorescence microscope.

### Immunofluorescence staining

Cells were fixed with 4% paraformaldehyde (PFA) for 10–15 min and washed with PBS for 3 times and then blocked with 3% BSA for 30 min. Cells were incubated with primary antibodies (LC3 and LAMP1, 1:100, Abcam) at 4 °C overnight. Afterward, they were washed with PBS and incubated with second antibody donkey anti-rabbit conjugated with Alexa Fluor 488 (1:400) and donkey anti-mouse conjugated with Alexa Fluor 594 (1:200) (all from Thermo Fisher) for 1 h at room temperature. Cell nuclei were stained with DAPI (Sigma), and the images were visualized using an inverted fluorescence microscope. ImageJ was employed for the quantification analysis of images.

### Western bolt

UMSCs were lysed in RIPA lysis buffer (Thermo Fisher Scientific), and total protein concentration was measured with BCA protein assay kit (Thermo Fisher Scientific). The PVDF membrane (Invitrogen) transferred protein was blocked with 5% non-fat milk and then incubated with primary antibodies overnight at 4 ℃. After washing with TBST for three times, the PVDF membrane was incubated with corresponding HRP-conjugated second antibody (1:1000, Cell Signaling Technology) at room temperature for 1 h. The primary antibody information is as follows, Bcl-2 (1:1000, abclonal), cleaved Caspase-3 (1:1000, abclonal), glutathione peroxidase 4 (GPX4, 1:1000); hexokinase 1 (HK1, 1:1000); hexokinase 2 (HK2, 1:1000); phosphofructokinase (PFK, 1:1000); lactate dehydrogenase A (LDHA, 1:1000); citrate synthase (CS, 1:1000); aconitase 2 (ACO2, 1:1000); fumarase (FH, 1:1000); succinate dehydrogenase A (SDHA, 1:1000); all were obtained from Abcam.

### Metabolite analysis

When UMSCs were cultured up to 90% confluency, the cells or cell supernatant was collected for metabolite analysis. To collect cell supernatant, the cells were rinsed with PBS and cultured in the basic medium for 24 h at 37 °C. Afterward, the cell supernatant was collected to analyze metabolites for energy metabolism. As for cell supernatant, the cell supernatant mixed with 1 mL of cold methanol/acetonitrile/H_2_O (2:2:1, v/v/v) was sonicated at a low temperature and were then incubated at − 20 °C for 1 h, followed by centrifugation for 15 min (14,000 g, 4 °C). The supernatant was dried in a vacuum centrifuge and stored at − 80 °C. As for cell lysate, the cells were washed with PBS for 3 times and then collected cells with a cell scraper, and the cell were pelleted by centrifuging for 5 min (1000 rpm, 4 °C), and snap-frozen in liquid nitrogen. A homogenate of 50 mg sample mixed with 1 mL of cold methanol/acetonitrile/H_2_O (2:2:1, v/v/v) was sonicated at a low temperature (30 min/ once, twice) and was incubated at − 20 °C for 1 h, followed by centrifugation for 20 min (14,000 g, 4 °C). The supernatant was dried in a vacuum centrifuge. Sextuplicate samples were collected and sent to analyze metabolites by Metabolon-associated energy metabolism (Applied Protein Technology, Shanghai, China). For LC–MS analysis, the dried samples were dissolved in 100 µL acetonitrile/H_2_O (1:1, v/v), adequately vortexed, and then centrifuged (14,000 rpm, 4 °C, 15 min). The supernatants were collected for the LC–MS/MS analysis. The samples were separated by Agilent 1290 Infinity LC ULTRA performance liquid chromatography system. Then, A 5500 QTRAP mass spectrometer (MS, AB SCIEX) was used to analyze the chromatographic peak area and retention time in anion mode. The energy metabolite standard was used to correct the retention time and identify metabolites.

To test glucose uptake and lactate secretion in UMSCs, we cultured UMSCs up to 80% confluency. The cells were changed to fresh medium with 5% UltroGRO and were cultured for 24 h, and then the concentration of glucose and lactate was measured by Glucose kit and Lactate kit (Cedex Bio), respectively,  using Cedex Bio biochemical analyzer (Roche).

### Transcriptomics sequence

Total RNAs of UMSCs were extracted using RNA Isolation Kit (TIANGEN, China). Agient 2100 Bioanalyzer Agilent and LabChip GX (PerkinElmer) were used to qualify and quantify RNA samples. dsDNA library was constructed using VAHTS Universal V6 RNA-seq Library Prep Kit (Vazyme, China) and was purified using VIHTS DNA Clean Beads (Vazyme, China) according to the manufacturer’s instruction. Qsep-400 and Qubit TM dsDNA assay kit were used to qualify and quantify dsDNA library. The qualified dsDNA samples were subsequently sequenced using Illumina Nova Seq 6000 platform (San Diego, USA). Raw sequencing reads were mapped to GRCh38 assembly of the human genome using Tophat2, version 2.0.10 [27]. Fragments per kilobase of exon model per million mapped reads (FPKM) was computed using Stringtie and normalized with TMM [28–30]. Differentially expressed genes (DEGs) were analyzed using DESeq 2 package, version 1.10.1 [31]. Genes with log-fold change > 1.5 and false discovery rate (FDR) < 0.05 were considered significantly transcriptomic changes.

### Statistical analysis

Data are presented as the mean ± SEM derived at least three independent experiments. Statistical significances were tested by Student’s *t*-test, and *p* values ≤ 0.05 were considered statistically significant. The fluorescence intensity was quantified by ImageJ.

## Results

### Reduced clonogenicity ability and proliferation potential of UMSCs at P9

**Fig. 1:**
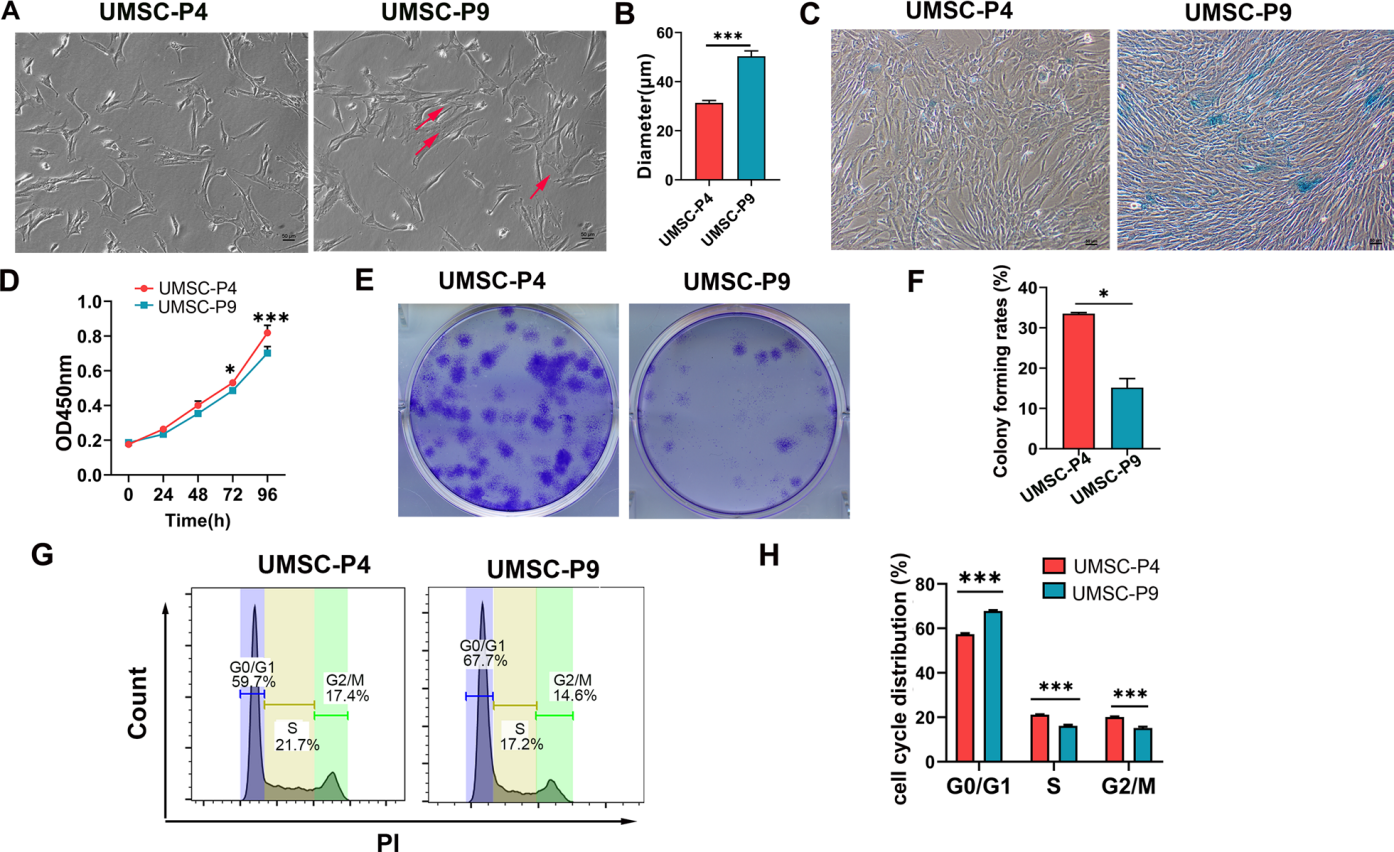
Cell morphology and the proliferation potential of UMSC-P4 and UMSC- P9. **a** Phase contrast images of UMSC-P4 and UMSC-P9 at day 1. Red arrows indicate flatten and larger size phenotype of cells. Scale bar, 50 µm. **b** Cell diameter of UMSC-P4 and UMSC-P9 at day 2 was measured by imageJ, at least 50 cells were calculated. **c** SA-β-gal staining of UMSC-P4 and UMSC-P9. Scale bar, 50 µm. **d** Cell proliferation potential of UMSC-P4 and UMSC-P9 was tested by CCK-8 kit (n ≥ 3). **e** Representative images of colony forming assay of UMSC-P4 and UMSC-P9 after cultured for 10 days. **f** Quantitative analysis of colony forming rate of UMSC-P4 and UMSC-P9 (n ≥ 3). **g** Representative images of cell cycle distribution in UMSC-P4 and UMSC-P9. **h** Quantification of cell cycle distribution of UMSC-P4 and UMSC-P9 (n ≥ 3). *P < 0.05 and ***P < 0.001 compared to UMSC-P4

We firstly assessed the phenotype and proliferation ability of UMSCs at P4 (UMSC-P4) and P9 (UMSC-P9). Figure 1a–c show that many cells in UMSC-P9 displayed a flat morphology, enlarged cell size and diameters, as well as stronger β-gal staining, a senescent phenotype than UMSC-P4. Moreover, UMSC-P9 displayed a reduced proliferation ability compared to UMSC-P4 (Fig. 1d). Next, we studied the colony-forming capacity of UMSCs. We found that under low-density culture conditions, the number of formed colonies in UMSC-P9 was far less than that of UMSC-P4 (Fig. 1e, f). We then further analyzed the cell cycle distribution of UMSCs and found that most cells in UMSC-P4 and UMSC-P9 rested at the G0/G1 phase. However, compared to UMSC-P4, the proportion of UMSC-P9 in G0/G1 phase was higher and that in G2/M phase was lower, indicating that more cells in UMSC-P9 were arrested in G0/G1 phase (Fig. 1g, h). As for cell surface markers, the expression pattern of UMSC-P9 was similar to that of UMSC-P4 (Additional file 1: Fig. S1). Therefore, these results indicated that UMSCs after long-time culture in vitro had a lower clonogenicity capacity and proliferation ability and displayed a senescent morphology.

### Decreased biological activity of UMSCs after long-time culture

**Fig. 2:**
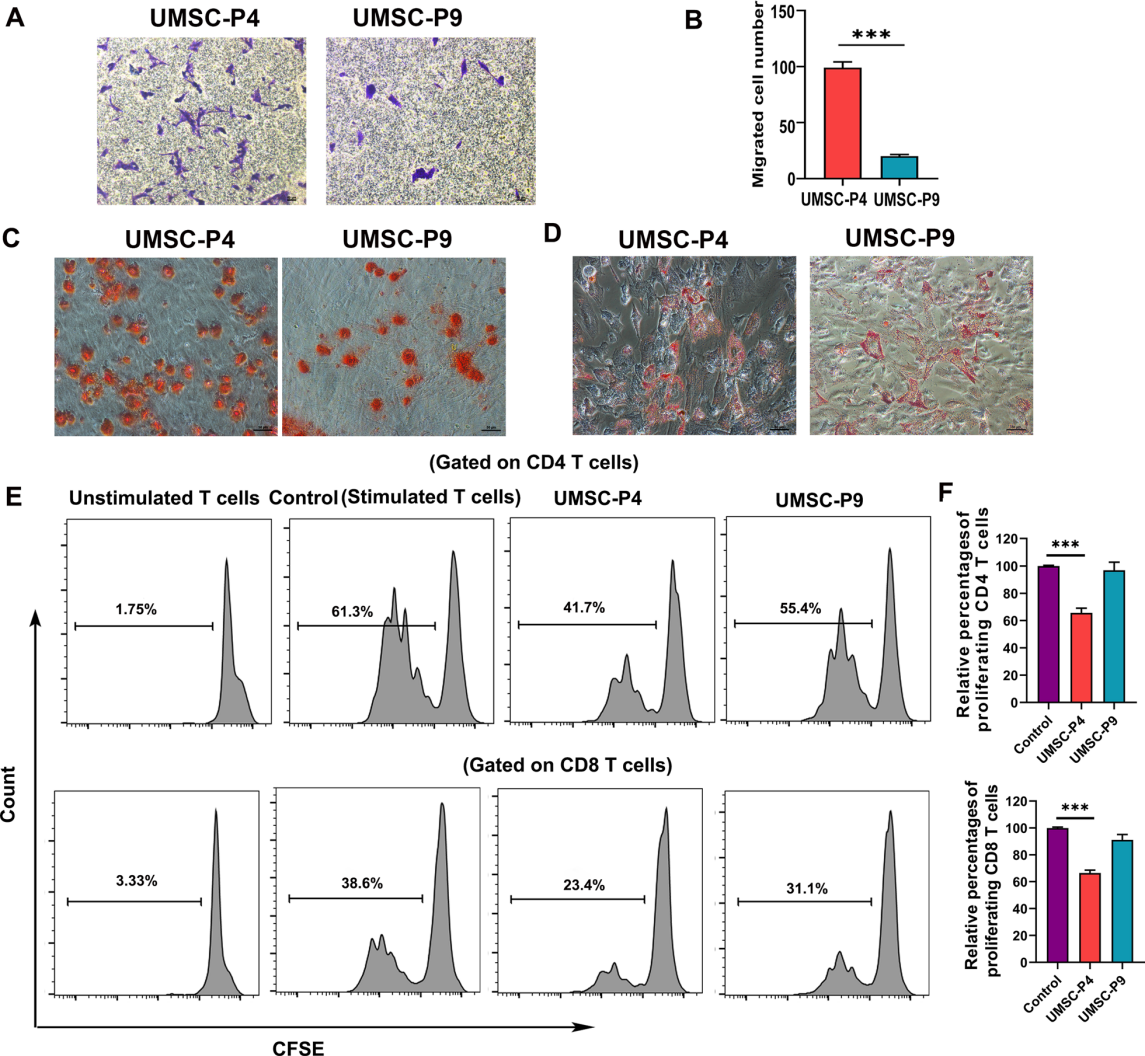
The biological activity of UMSC-P4 and UMSC-P9. **a** Representative images of Transwell migration assays of UMSC-P4 and UMSC-P9. Scale bar, 50 µm. **b** Quantification of migrated cell numbers in UMSC-P4 and UMSC-P9. At least 20 representative visual fields for each group were counted. **c** UMSCs were differentiated into osteoblasts for 15 days and then analyzed by alizarin red staining. Scale bar, 50 µm. **d** UMSCs were differentiated into adipocytes for 20 days and then analyzed by oil red-O staining.  Scale bar, 50 µm. **e, f** Inhibitory effects of UMSCs on the proliferation of CD4+ T cells and CD8+ T cells, **e** Representative images of flow cytometry of proliferating CD4+ T cells and CD8+ T cells in different groups. Unstimulated T cells have a single peak at a high fluorescent. Upon stimulated, lower fluorescent intensity peaks form. **f** Quantification of the relative percentage of proliferating CD4+ T cells and CD8+ T cells in different groups (n ≥ 3). The relative percentages of CD4+ T cells and CD8+T cells in control group were set as 100%. ***P < 0.001 compared to control

Increasing evidence suggests that MSCs exhibit therapeutic effects due to the following features: First, MSCs can migrate to damaged and/or diseased sites due to homing ability [32]. Secondly, MSCs can repair injured and/or diseased tissues due to their paracrine function, differentiation capacity, and immunomodulatory ability [33]. Here, we compared these biological activities of UMSC-P4 and UMSC-P9. First, we assessed the migratory ability of UMSCs with Transwell assays. UMSCs were seeded in the upper chamber with UltroGro-free α-MEM medium, and α-MEM medium with 5% UltroGro was added in the lower chamber. We found that UMSC-P9 displayed reduced mobility with a smaller number of migrated cells than that of UMSC-P4 (Fig. 2a, b). To measure the differentiation potential of UMSCs, UMSC-P4 and UMSC-P9 were induced to differentiate into osteocytes or adipocytes in the corresponding differentiation medium for 15–25 days. Then, the osteogenic and adipogenic differentiation potential of UMSC-P4 and UMSC-P9 were measured by alizarin red and oil red staining, respectively. Fewer differentiated bone-like nodules were observed in UMSC-P9 compared to those of UMSC-P4 (Fig. 2c), although UMSC-P4 and UMSC-P9 had a similar number of adipose globules (Fig. 2d). The phenomena were consistent with our previous study [4]. Then, the immunosuppressive capacity was evaluated. Previous studies reported that MSCs could inhibit the proliferation of lymphocyte, including CD4 and CD8 T cells [4, 34]. To test the immunosuppressive capacity of UMSCs, we first labeled PBMCs with CSFE, then co-cultured them with UMSCs at a ratio of 5:1 for 3 days. Finally, we harvested the above PBMCs and performed flow cytometry analysis. The percentages of the proliferation of CD4 T cells and CD8 T cells were increased in the control group without UMSCs. However, UMSC-P4 could inhibit the proliferation of CD4 T cells and CD8 T cells with an inhibitory rate of 34.12% ± 1.49% and 33.43% ± 2.2%, respectively, while UMSC-P9 had no inhibition function on CD4 T cells and CD8 T cells (Fig. 2e, f). Taken together, these results confirmed that UMSCs at late passages exhibited a low proliferation ability and weak biological activities including homing ability, differentiation potential, and immunosuppressive capacity.

### Changes of mitochondrial morphology and membrane potential of UMSCs after long-time culture in vitro

**Fig. 3:**
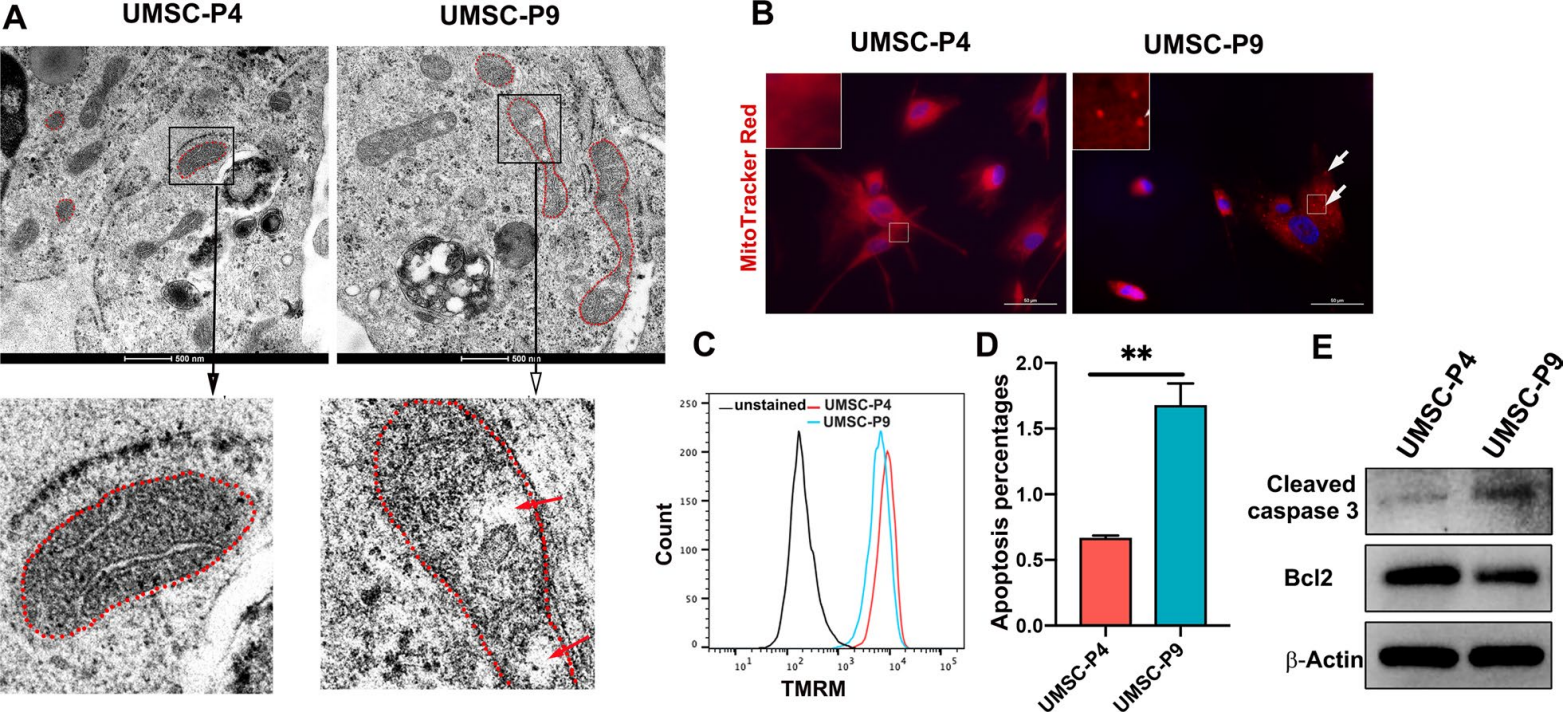
The mitochondrial morphology of UMSC-P4 and UMSC-P9. **a** Electron microscope images show mitochondrial morphological and structure changes in UMSC-P4 and UMSC-P9. Mitochondria of UMSC-P9 became larger and longer than that of UMSC-P4. Red dotted circles represent mitochondria. The mitochondrial structure of UMSC-P4 were clear and well-organized. In contrast, that of UMSC-P9 were unclear and disordered, and some swollen regions devoid of cristae (red arrow). Scale bar, 500 nm. **b** Representative images of the mitochondria of UMSC-P4 and UMSC-P9 stained by MitoTracker Red. Some small and punctate fragments (white arrow) were visible in the mitochondria of UMSC-P9. Scale bar, 50 µm. **c** Representative flow cytometry images of the membrane potential of UMSC-P4 and UMSC-P9 revealed by TMRM probe. **d** Quantification of apoptosis percentages in UMSC-P4 and UMSC-P9 (n ≥ 3). **e** Western Blot analysis of cell apoptosis of UMSC-P4 and UMSC-P9 with the antibodies against cleaved caspase 3, Bcl2 and β-actin. The protein level of β-actin was used as internal control

Mitochondria are cellular organelles responsible for cellular metabolism, ATP production, redox homeostasis, calcium homeostasis, iron metabolism, and regulation of intrinsic apoptosis and ferroptosis [11–13]. To investigate the morphological and functional changes of UMSCs at late passages, we first compared mitochondrial morphology, mitochondrial membrane potential and cell apoptosis of UMSC-P4 and UMSC-P9. We examined the mitochondrial structure of UMSCs using transmission electron microscope (TEM) and found that the mitochondrial size of UMSC-P9 was generally larger and longer than that of UMSC-P4, indicating that many mitochondria of UMSC-P9 could be swollen. The mitochondria of UMSC-P4 displayed a clearly defined and well-ordered structure. In contrast, many mitochondria of UMSC-P9 containing fewer cristae exhibited less clear and disordered structure, and some distinct swollen regions contained no cristae at all (arrow) (Fig. 3a). Next, we stained the mitochondria of UMSCs with MitoTracker Red. Figure 3b shows that small punctate fragments were apparent in the mitochondria of UMSC-P9. At the same time, the mitochondrial distribution of UMSC-P4 was continuous and uniform. Mitochondria are highly dynamic and have branching interconnected networks, which are controlled by the balance of fusion and fission [35]. Increasing studies indicated that global mitochondrial fragmentation (mitochondrial fission) is a pathological phenotype, which can lead to cell apoptosis and cell death [36, 37]. Currently, it is accepted that the integrity of the mitochondrial structure is crucial for correct mitochondrial function, especially cristae structure integrity [38]. All the above results reveal that mitochondrial structure and dynamics of UMSCs at late passage are impaired and could affect mitochondrial function.

Next, we measured the membrane potential of UMSCs using tetramethylrhodamine methyl ester (TMRM) dye, a cation mitochondrial selective probe. Flow cytometry analysis showed that UMSC-P9 had a lower membrane potential (Fig. 3c). Previous reports have demonstrated that mitochondria take part in apoptosis and that disturbed mitochondria dynamics promotes the release of cytochrome C and caspase activation [39]. This apoptosis process can be inhibited by the antiapoptotic protein Bcl2 [39]. Next, we assessed the apoptosis of UMSC-P4 and UMSC-P9 with PI and Annexin V kit by flow cytometry. Additional file 2: Fig. S2a and Fig. 3d shows that the percentage of apoptotic cells was increased in UMSC-P9 compared to that of UMSC-P4. We further analyzed the protein levels of cleaved caspase 3 and Bcl2 by western blot and found that the level of cleaved caspase 3 was higher and the level of Bcl2 was lower in UMSC-P9 compared to UMSC-P4 (Fig. 3e). Thus, these results indicated that the mitochondria dynamics of UMSCs at late passage that was disturbed could cause increased cell apoptosis.

### Decreased response to oxidative stress and increased ferroptosis of the mitochondria in UMSCs after long-time culture in vitro

**Fig. 4:**
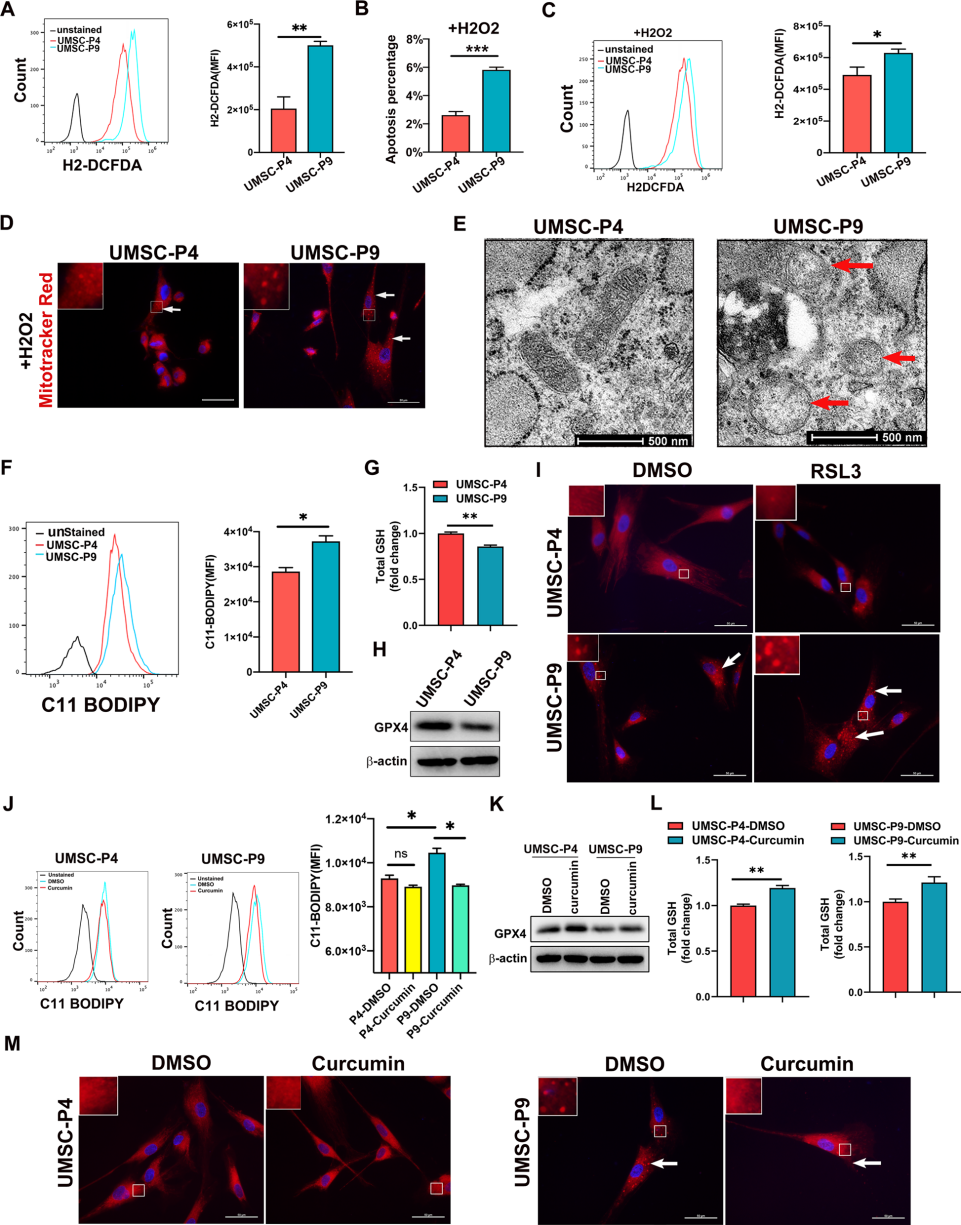
Decreased response to oxidative stress and increased ferroptosis of the mitochondria in UMSC-P9. **a** Representative flow cytometry image of ROS levels detected by H2-DCFDA and Quantification of ROS levels in UMSC-P4 and UMSC-P9 (n ≥ 3). **b** Quantification of apoptosis percentages of UMSC-P4 and UMSC-P9 treated with H_2_O_2_ (n ≥ 3). **c** Representative flow cytometry image of ROS levels and quantification of ROS levels in UMSC-P4 and UMSC-P9 treated with H_2_O_2_ (n ≥ 3). **d** Representative mitochondrial images of UMSC-P4 and UMSC-P9 treated with H_2_O_2_ stained with MitoTracker Red. Scale bar, 50 µm. **e** Representation of TEM images of the mitochondria of UMSC-P4 and UMSC-P9. The red arrow indicates the mitochondrion with the reduction and disappearance of mitochondrial cristae. **f** Representative flow cytometry images of lipid ROS levels and quantification of lipid ROS levels in UMSC-P4 and UMSC-P9 (n ≥ 3). **g** Total GSH levels in the cell lysate in UMSC-P4 and UMSC-P9 (n ≥ 3). The concentration of UMSC-P4 was set as 1. **h** Western Blot analysis of GPX4 levels in UMSC-P4 and UMSC-P9. **i** Representative mitochondrial images of UMSC-P4 and UMSC-P9 stained with MitoTracker Red after treated with 5 µM RSL3 for 24 h. Scale bar, 50 µm. **j** Representative flow cytometry images of lipid ROS levels and quantification of lipid ROS levels in UMSC-P4 and UMSC-P9 after treated with 2 µM curcumin for 48 h (n ≥ 3). **k** GPX4 levels were detected in UMSC-P4 and UMSC-P9 by western blot. **l** Total GSH levels in the cell lysate in UMSC-P4 and UMSC-P9 (n ≥ 3). The concentration of UMSC-P4 and UMSC-P4 treated with DMSO was set as 1, respectively. **m** Representative mitochondrial images of UMSC-P4 and UMSC-P9 stained with MitoTracker Red after treated with curcumin. Scale bar, 50 µm. White arrows represent punctate fragments of mitochondria

Previous studies reported that the mitochondrial fragmentation occurs in response to oxidative stress, and antioxidants prevent mitochondrial fragmentation caused by excessive oxidative stress [40]. Therefore, we measured the levels of reactive oxygen species (ROS) of UMSC-P4 and UMSC-P9 with an oxidant-sensing fluorescent probe H2-DCFDA. As shown in Fig. 4a and Additional file 2: Fig. S2b, UMSC-P9 produced more ROS than UMSC-P4. In this regard, the mitochondrion is an organelle of ROS production and scavenging, as it contains many antioxidative enzymes and antioxidants [41]. Moreover, our previous study has demonstrated that UMSCs can decrease oxidative stress and oxidative damage [36]. Therefore, we wondered whether the long-time culture of UMSCs would decrease their response to excess ROS levels resulting in decreased antioxidative potential. We treated UMSC-P4 and UMSC-P9 with 50 µM H_2_O_2_ for 24 h, then examined cell viability and ROS levels. As shown in Fig. 4b, Additional file 2: Fig. S2c and d, there were more apoptotic cells in H_2_O_2_-treated UMSC-P9 than those in UMSC-P4. Furthermore, UMSC-P9 contained higher ROS levels than UMSC-P4 (Fig. 4c and Additional file 2: Fig. S2e). Next, we stained H_2_O_2_-treated UMSCs with MitoTracker Red and found that excess ROS damaged the mitochondria morphology. Although punctate mitochondrial fragments were observed in both UMSC-P4 and UMSC-P9, the number and size of mitochondrial fragments of UMSC-P9 were more and bigger (Fig. 4d). Thus, these results suggested that long-time culture in vitro of UMSCs induced more ROS production and reduced antioxidative capacity, leading to mitochondrial fragmentation, cell apoptosis and death.

Mitochondrion is also an organelle that plays a crucial role in iron metabolism, associated with oxidative stress and ferroptosis [42, 43]. To investigate whether long-time culture in vitro of MSCs can cause ferroptosis, we first observed the morphological features of UMSC-P9 by TEM. Figure 4e shows that some mitochondria of UMSC-P9 characterized by severely disrupted and even disappeared crista in UMSC-P9 (red arrow). As ferroptosis is iron-dependent lipid peroxidation, lipid ROS levels are regarded as a method to determine cell ferroptosis. Next, we measured the lipid ROS level of UMSC-P4 and UMSC-P9 with C11 BODIPY, a lipid peroxidation sensor. As shown in Fig. 4f, the fluorescence intensity of UMSC-P9 was higher than that of UMSC-P4, indicating long-time culture in vitro of UMSCs could induce more lipid ROS generation and ferroptosis in UMSCs. Normally, GPX4 is only a GSH peroxidase or thiol peroxidase enzyme, which can catalyze the reduction of lipid peroxides in a cellular membrane environment [44]. Therefore, we tested the levels of GPX4 and GSH in the cell lysate. Western blot assay showed that the level of GPX4 was reduced in UMSC-P9 compared to that of UMSC-P4 (Fig. 4g). Total cellular GSH level in UMSC-P9 was also decreased (Fig. 4h). These data suggested that the ferroptosis of UMSC-P9 are increased. We next wondered whether increased ferroptosis could induce mitochondrial damage. We treated UMSC-P4 and UMSC-P9 with 5 mM RSL3, an activator of ferroptosis, for 24 h, and found that RSL3 induced more mitochondrial fragments in UMSC-P9 (Fig. 4i), while fewer mitochondrial fragments were appeared in UMSC-P4. Besides, we wanted to treat UMSCs with ferroptosis inhibitors. Previous study reported that curcumin, a phenolic antioxidant, can ameliorate erastin-mediated ferroptosis in MIN 6 pancreatic beta cell [45]. Moreno et al. [46] found that curcumin reduces ferroptosis and ferroptosis-mediated renal damage in rhabdomyolysis-associated renal damage model. Thus, we used 2 mM curcumin to treat UMSCs for 48 h. We found that curcumin remarkably reduced lipid ROS level in UMSC-P9 and increased the levels of GPX4 and GSH in UMSC-P4 and UMSC-P9 (Fig. 4j–l). Moreover, curcumin evidently reduced mitochondrial fragments generation in UMSC-P9 (Fig. 4m). The data demonstrated that curcumin could inhibit ferroptosis in long-time culture of UMSC and UMSC-P9 is more sensitive to ferroptosis activator and inhibitor. Conclusively, the above results indicated that the long-time culture of UMSCs induced more ROS production, including lipid ROS, and a decrease of antioxidative potential, resulting in increased ferroptosis and mitochondrial fragmentation.

### Reduction in the energy metabolism of UMSC after long-term culture in vitro

**Fig. 5:**
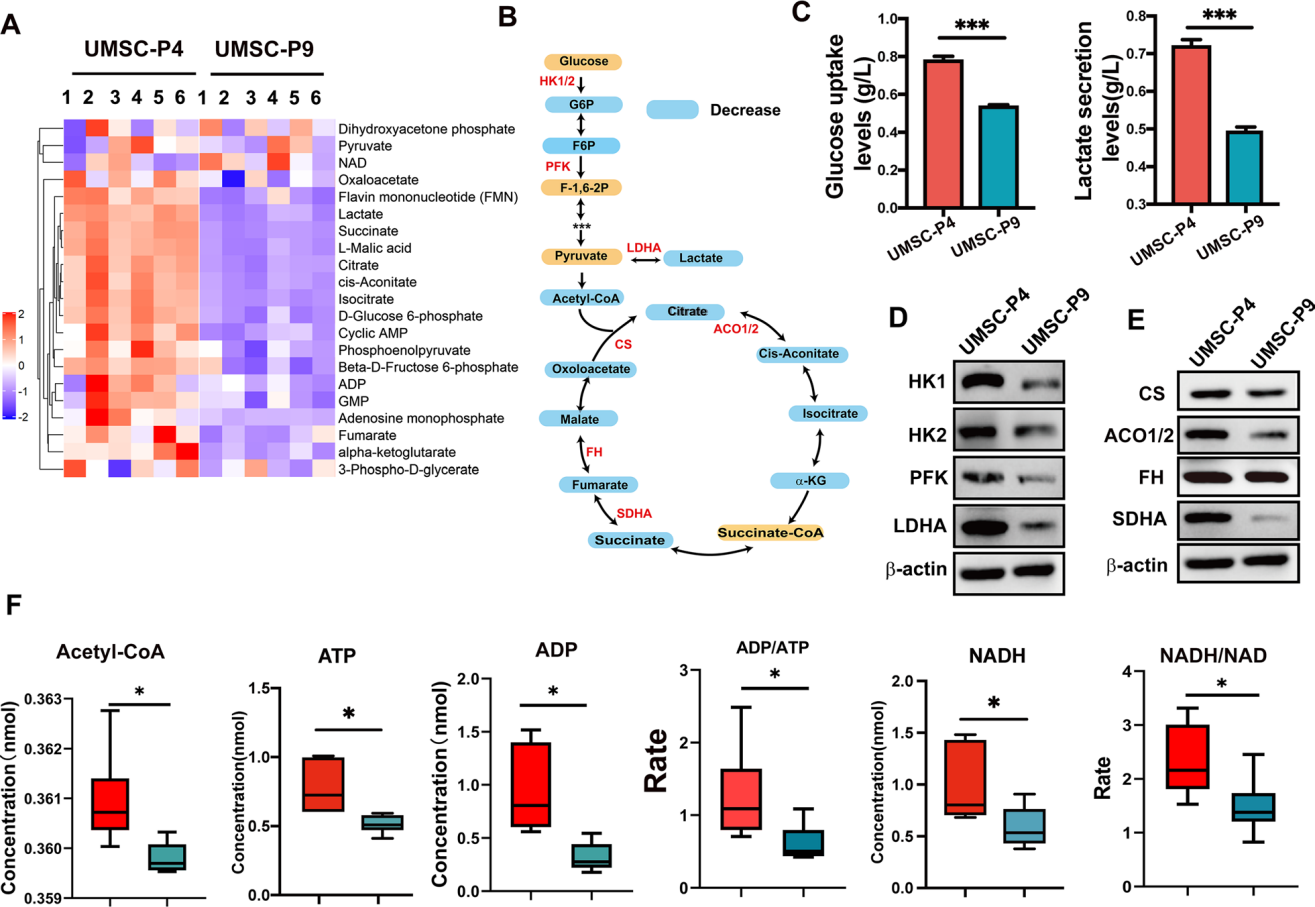
Changes of metabolites and enzymes in glycolysis and TCA in UMSCs after long-term culture in vitro. **a** Heatmap of significant up- and down-regulated metabolites in cell supernatant in UMSC-P9 versus UMSC-P4. **b** schematic diagram of intermediate metabolites of glycolysis pathway and TCA in the supernatant of UMSC-P4 and UMSC-P9 are shown in the histogram (n = 6). Significant downregulated were shown with the blue box. **c** The concentration of glucose uptake and produced lactate levels in the supernatant produced in UMSC-P4 and UMSC-P9 after culture for 24 h (n ≥ 3). **d, e** Western blot analysis of glycolysis-related enzymes (d) and TCA-related enzymes: hexokinase 1: HK1; hexokinase 2: HK2; phosphofructokinase: PFK; lactate dehydrogenase A: LDHA; citrate synthase: CS; aconitase 2: ACO2; fumarase: FH; succinate dehydrogenase A:SDHA, **e** in UMSC- P4 and UMSC-P9. **f** Levels of acetyl-CoA, ATP, ADP, NADH, and the rates of ADP/ATP and NADH/NAD in the cell lysates of UMSC-P9 and UMSC-P4 (n = 6). *P < 0.05, ***P < 0.01 compared with UMSC-P4

Mitochondria are the main site for ATP synthesis through the tricarboxylic acid (TCA) cycle and oxidative phosphorylation (OXPHOS) [47]. To investigate the change of energy metabolism in mitochondria of UMSCs after long-time culture, we performed energy metabolites profiling of UMSC-P4 and UMSC-P9 (n = 6), which targets 32 metabolites, including the metabolites of glycolytic pathway, TCA and OXPHOS process. There are  thirty-two energy metabolites measured with cell supernatant or cell lysate of UMSCs by using high-performance liquid chromatography/mass spectrometry (HPLC/MS) and gas chromatography/MS (GC/MS) according to the protocol described in the Materials and Methods. Among these metabolites, twenty-two metabolites in cell supernatant were identified and attained a relative level of these metabolites (Additional file 4: Table S1). The heat map of cell supernatant of UMSC-P4 and UMSC-P9 shows that the profiles of energy metabolites were different between UMSC-P4 and UMSC-P9 (Fig. 5a). Interestingly, sixteen metabolites were significantly downregulated in UMSC-P9 (*P* < 0.05), including metabolites from glycolysis (glucose 6-phosphate, G6P; fructose 6-phosphate, F6P; fructose 1,6-bisphosphate, F-1,6-2P, lactate), TCA cycle and OXPHOS process (citrate, cis-Aconitate, isocitrate, α-ketoglutarate, α-KG, succinate, fumarate, malate, oxaloacetate, ADP, FMN), and nucleotide synthesis (AMP, cAMP, GMP) (Fig. 5a, b and Additional file 3: Fig. S3). To verify energy metabolism changes, including the glycolytic pathway and TCA, we measured the glucose uptake and lactate production levels in the supernatant 24 h after culturing in a fresh medium and found that the glucose uptake and lactate production were decreased in UMSC-P9 (Fig. 5c). These results indicated that the glycolytic pathway and TCA cycle could be downregulated in UMSCs after long-time culture. We further performed western blot analysis with the enzymes involved in the glycolytic pathway (HK1/2, PFK, ALDHA) and TCA (CS, ACO1/2, FH, SDHA) and found their expression levels were decreased in UMSC-P9, indicating the activity of glycolytic pathway and TCA cycle in UMSC-P9 were reduced (Fig. 5d, e).

Subsequently, we further measured the intracellular energy metabolites of UMSC-P4 and UMSC-P9 using HPLC/MS and GC/MS. We found that the levels of acetyl-CoA, ATP, ADP, and NADH, which drive OXPHOS to produce ATP, and the rates of ADP/ATP and NADH/NAD were significantly downregulated in UMSC-P9 (*P* < 0.05) (Fig. 5f and Additional file 5: Table S2). In conclusion, some metabolites of the TCA cycle, TCA-related enzymes, NADH, ADP, ATP were downregulated in UMSC-P9, suggesting that the activities of TCA and OXPHOS in UMSC-P9 were decreased, and the function of energy metabolism of mitochondria was impaired in UMSCs after long-time culture in vitro.

### Impaired lysosomal function in UMSCs after long-time culture

**Fig. 6:**
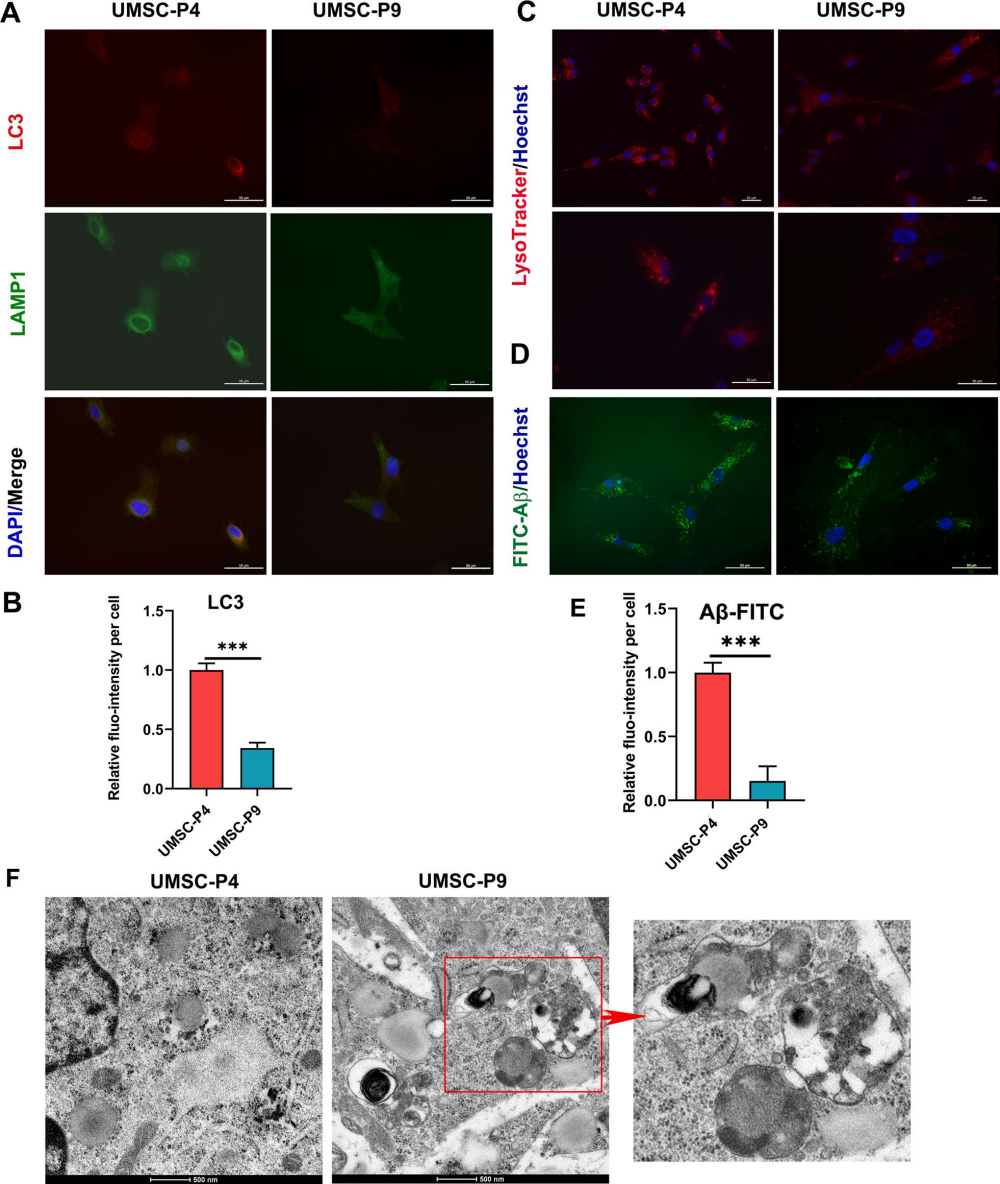
Lysosomal morphology and function were impaired in UMSC-P9. **a** Representative image of autophagy in the lysosomes of UMSC-P4 and UMSC-P9 by immunofluorescence staining with the antibodies against LC3 and LAMP1. Scale bar, 50 µm. **b** Quantification of fluorescence (fluo-) intensity of LC3 per cell in UMSC-P4 and UMSC-P9. At least 50 cells in each group were tested. **c** Representative images of lysosomal morphology and distribution in UMSC-P4 and UMSC-P9 stained with lysoTracker Red. Scale bar, 50 µm. **d** Phagocytosis was measured by phagocytic Aβ-FITC in each cell of UMSC-P4 and UMSC-P9 after treated with Aβ-FITC for 4 h. Scale bar, 50 µm. **e** Qualification of fluorescence (fluo-) intensity of Aβ-FITC of per cell in UMSC-P4 and UMSC-P9. At least 50 cells in each group were measured. **f** Electron microscope images of UMSC-P4 and UMSC-P9 show morphological changes of lysosomes. The red box represents atypical lysosomes with some undegraded materials. Scale bar, 500 nm

Damaged or dysfunctional mitochondria are eliminated by an autophagic system, termed as mitophagy that contributes to maintaining mitochondrial quantity and quality[48]. To investigate the autophagy of UMSCs after long-time culture, we performed co-immunofluorescence with LC3, a marker of autophagosomes, and lysosome-associated membrane glycoprotein 1 (LAMP1), a major lysosomal membrane protein. Figure 6a and b shows that LC3 in UMSC-P4 displayed a strong fluorescence with a perinuclear distribution, while LC3 in UMSC-P9 displayed a weak fluorescence with a diffuse cytoplasmic distribution. In addition, LAMP1 staining displayed a similar pattern and colocated with LC3. This result indicated that long-time culture UMSCs had a declined autophagy ability, including mitophage capacity. Different forms of stress are shown to induce lysosomal membrane permeabilization, LAMP1 and LAMP2 have been reported to protect the membrane from degradation by lysosomal enzymes [16, 49]. As shown in Fig. 6a, the diffuse cytoplasmic distribution of LAMP1 observed in UMSC-P9 implied an increase  of lysosomal membrane permeabilization of UMSCs after long-time culture. To confirm this hypothesis, we performed a LysoTracker red, a specific dye for lysosome staining. Diffuse cytoplasmic distribution of LysoTracker red was observed in the UMSC-P9, while the perinuclear accumulated distribution of lysosome was visible in UMSC-P4 (Fig. 6c). In addition, lysosomes can degrade extracellular macromolecules by phagocytosis [50]; therefore, we cultured UMSCs with FITC-beta-amyloid (Aβ-FITC) to test the lysosomal phagocytic capacity of UMSCs. As shown in Fig. 6d and e, there were more FITC-Aβ in the cytoplasm of UMSC-P4 than UMSC-P9, indicating a decline of phagocytic capacity in UMSC-P9. To provide additional evidence that there is a change in the lysosomal morphology of UMSC-P9, we performed electronic microscopy. As shown in Fig. 6f, many enlarged lysosomes contained undegraded materials in UMSC-P9 (red box), indicating a decline of the lysosomal ability of degradation in UMSCs aging in vitro. To this end, the above results revealed that the lysosomal activity was decreased, and the function of lysosomes was impaired in UMSCs after long-time culture.

### Transcriptome analysis of UMSC-P4 and UMSC-P9

**Fig. 7:**
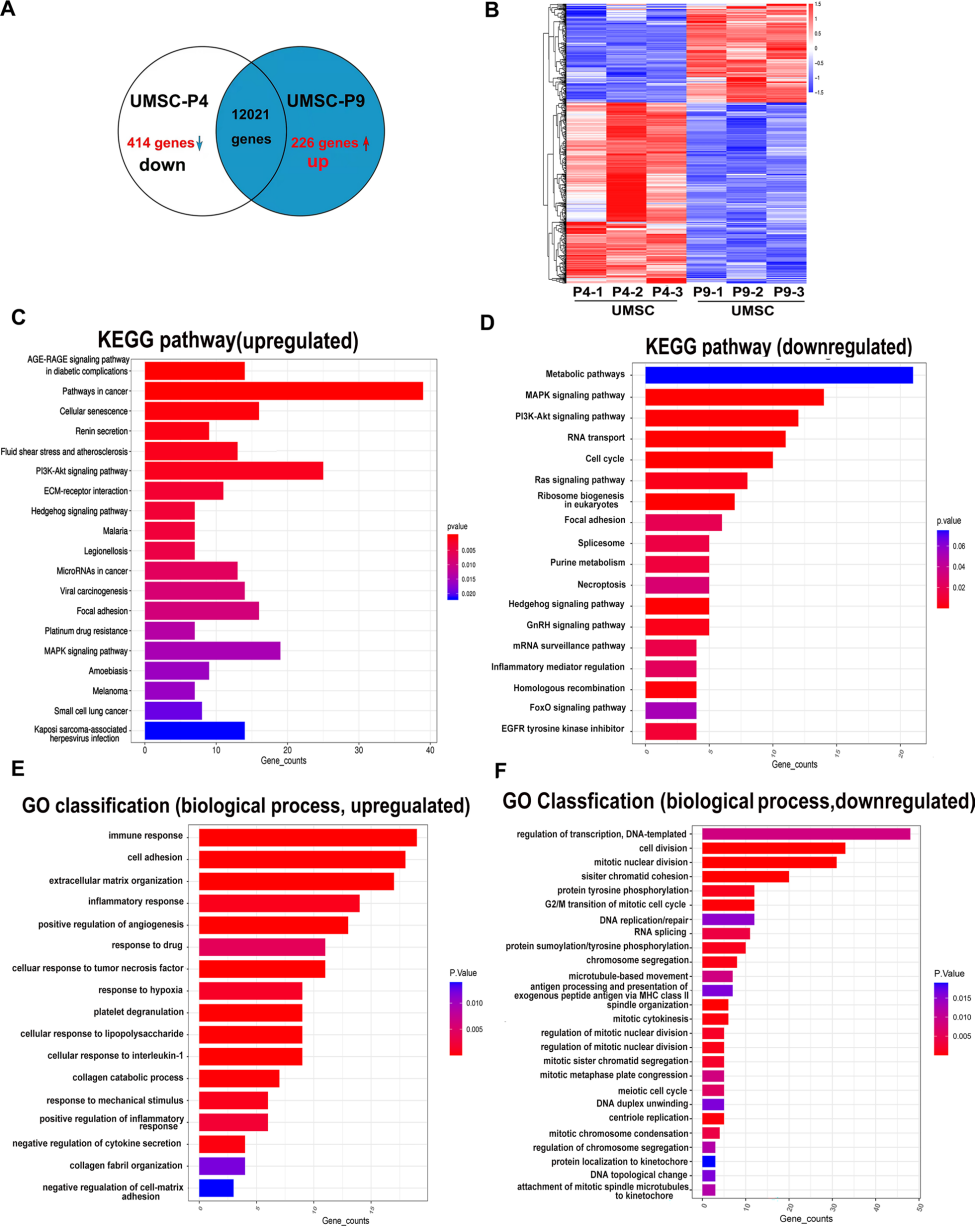
Transcriptome analysis of UMSC-P4 and UMSC-P9. **a** The Venn diagram depict the entire transcriptome of tested gene transcripts in UMSC-P9 and UMSC-P4. The numbers of transcripts with significant gene expression changes in UMSC-P9 versus UMSC-P4 (1.5-fold differential expression and statistical significance, *p*-value ≤ 0.05). **b** Heat map of differentially expressed gene expression of UMSC-P9 and UMSC-P4 (n = 3). **c, d** KEGG pathway enrichment analysis of the upregulated and downregulated pathways in UMSC-P9 versus UMSC-P4. **e**, **f** Gene ontology (GO) classification analysis of the upregulated and downregulated gene changes in biological process in UMSC-P9 versus UMSC-P4

To determine the gene expression profiles of UMSCs after long-time culture, we compared the transcript profiles (total 12,021 transcripts) of UMSC-P4 and UMSC-P9 by RNA sequence (Additional file 6: Table S3). In general, the expression of many genes of UMSC-P4 and UMSC-P9 was similar. According to the multiple of gene differences (fold change ≥ 1.5) and statistical significance analysis (*p*-value ≤ 0.05), 226 transcripts were upregulated, and 414 transcripts were downregulated, respectively, in UMSC-P9 compared to those in UMSC-P4 (Fig. 7a, Additional file 7: Tables S4 and Additional file 8: S5). Moreover, differentially expressed genes were represented with a heatmap (Fig. 7b). Next, the genes of UMSC-P9 showing a more than 1.5-fold alteration were subjected to the KEGG pathway enrichment analysis and gene ontology (GO) classification function analysis. We found that the pathways related to senescence, cancers, infection, and diseases in UMSC-P9 were upregulated as compared to those in UMSC-P4 (Fig. 7c). However, the pathways related to cellular metabolism, cell cycle, cell divide, RNA transport, ribosome biogenesis, spliceosome, mRNA surveillance, homologous recombination, and FOXO signaling (antioxidative signaling) were downregulated in UMSC-P9 (Fig. 7d). The downregulated expression of metabolism-related genes in UMSC-P9 also confirmed the reduction of mitochondrial metabolism function (Fig. 5).

**Fig. 8:**
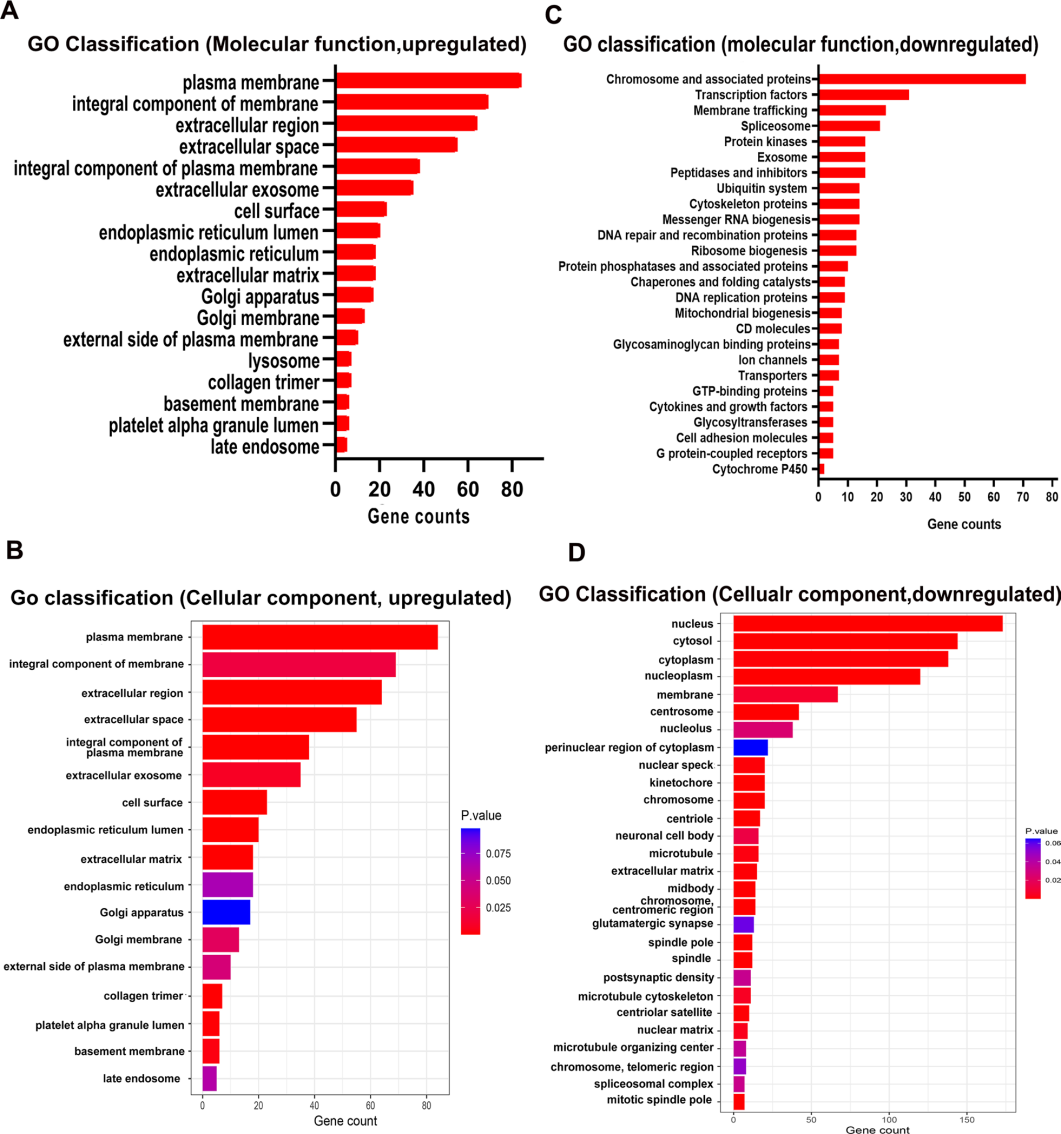
Gene ontology (GO) classification analysis of the upregulated and downregulated gene changes in molecular function and cellular component in UMSC-P9 versus UMSC-P4. Molecular function analysis (**a, c**). Cellular component analysis (**b, d**)

GO classification analysis indicated that the biological processes related to cell adhesion, immune response, inflammatory response, response to hypoxia, mechanical stimulus were upregulated (Fig. 7e). Moreover, the molecules related to receptor binding, cytokine activity, integrin binding, laminin binding, protease binding, MMP activity were upregulated (Fig. 8a). The data indicated that UMSC-P9 is more sensitive to oxygen pressure, mechanical stimulus, microenvironment, inflammation. Interestingly, the cellular component to endoplasmic reticulum, Golgi apparatus and membrane, endosome, which are organelles response to the changes of extracellular pressure, microenvironment, and immune response, were upregulated (Fig. 8b). However, the biological processes related to cell division, mitosis, microtubule-based movement, DNA repair/replication, message RNA biogenesis, and spliceosome were downregulated in UMSC-P9 (Fig. 7f). Moreover, the molecules related to chromosome and associated protein, transcription, protein kinase, mitochondrial biogenesis, ribosome biogenesis, cytoskeleton, chaperones, folding catalysts, ion channels, transports, cytokines, and growth factors were decreased in UMSC-P9 (Fig. 8c). These molecules are directedly associated with cell biological function and cell vitality. And more, the cellular components associated with nucleus, cytosol, cytoplasm, chromosome, centrosome, membrane, microtubule, and cytoskeleton were downregulated (Fig. 8d). The data indicated that the biological process related to cell division, cell proliferation, protein analysis, microtubules, synthesis of organelles, cell activity are decreased in UMSC-P9. Interestingly, as for mitochondria, the reduced expression of genes related to mitochondrial biogenesis proteins, cytoskeleton proteins and microtube-based movement in UMSC-P9 indicated an impaired mitochondrial morphology and function (Fig. 8c). As for lysosome, the ubiquitin system and chaperone play an essential role in ubiquitin-mediated selective autophagy [51] and chaperone-mediated autophagy chaperones [52]. Ion channels and transporter are important for maintaining ionic homeostasis in mitochondria and lysosomal lumen, such as Ca^2+^, Fe^2+^, H^+^ [22]. Therefore, the reduced expression of genes related to ubiquitin system, chaperones, ion channels, transports in UMSC-P9 indicated a disturbed lysosomal morphology and function (Fig. 8c). In short, the above analysis results indicated that the long-time culture UMSCs exhibited the decreased activities in cell metabolism, cell division, organelle biogenesis and degradation, cellular functions, resulting in cell cycle arrest and senescence, which eventually caused cell activity reduction.

## Discussion

In the present study, we compared mitochondrial morphology and function of UMSCs at an early passage and a late passage. Noteworthy, we found several senescent characteristics in UMSCs at late passages. First, we found mitochondria are rich in UMSCs (Fig. 3a, b), which is different from many types of stem cells, including embryonic stem cells [53], hematopoietic stem cells [54], neural stem cells [55]. The latter has a low abundance of mitochondria and an immature inner structure [56]. This difference implies an important function of mitochondria in UMSCs. Particularly, electron microscope examination showed that disordered mitochondrial structure and fewer crista in the mitochondria of UMSCs after long-time culture, provides important evidence for the aging phenomenon in UMSCs at late passages (Fig. 3a). Consistent with our finding, Marina et al. also reported the morphological changes in mitochondria in aging human umbilical vein endothelial cells [57]. In addition, small and punctate mitochondrial fragments appear in UMSC at late passage (Fig. 3b), which is considered as a phenomenon of mitochondrial fission, leading to the imbalance of fusion and fission activities and mitochondrial dynamics, and morphological  change [14, 58]. In this regard, our present study also shows that autophagy, including mitophagy is decreased in UMSCs at late passage. Moreover, the current GO analysis of the transcriptome of UMSCs showed that mitochondrial biogenesis and cytoskeleton proteins are downregulated (Fig. 8c). They are believed to play a crucial role in regulating mitochondrial dynamics and in controlling mitochondrial quality and quantity [59]. Therefore, these results of this study reveal that long-time culture of UMSCs induces mitochondrial dynamics impairment and morphological change.

Mitochondria are important organelles for energy metabolism and ATP production through the TCA cycle and OXPHOS [47]. Our findings showed that glucose uptake, NADH and ATP production, and many metabolites in TCA in late passage of UMSCs were decreased. In addition, ADP and ADP/ATP were higher in UMSC-P4 than those in UMSC-P9. Intriguingly, previous study demonstrated that in the presence of high ADP concentration, mitochondria are active with more ATP produced, while at low ADP concentration, mitochondria are inactive with less production of ATP [60]. Thus, we propose that the mitochondrion and TCA process may be active in early passaged UMSCs, and less active in the late passaged UMSCs. In this regard, it appears that the energy metabolism of UMSCs is different for many types of stem cells, which is dependent on glycolysis and a lower rate of oxidative phosphorylation for energy production [61]. Further supports for our claim come from the present study showing that UMSC-P9 exhibit a lower membrane potential, a higher ROS level, an increased ferroptosis and a reduced antioxidant potential than that of UMSC-P4 (Figs. 3 and 4). Therefore, current work suggests that mitochondrial dysfunction during UMSC aging in vitro probably contributes to the decline of stem cell activity, function, and therapeutic effects.

In addition to mitochondria, the present study also investigated lysosomal morphology and function of UMSCs aging in vitro. By performing electron microscopy, we demonstrated the presence of accumulated undegraded materials in some lysosomes, revealing a decline of lysosomal degradation ability in UMSCs aging in vitro, which echoes a pathogenic phenomenon in some lysosome storage and neurodegenerative diseases [62, 63]. Lysosomes degrade and recycle extracellular or intracellular macromolecules by endosomes or autophagosomes. In response to nutrient limitation and damage to proteins or organelles, eukaryotic cells initiate autophagy to maintain cellular energy balance and organelles homeostasis [21, 64]. Further support for the declined lysosomal activity comes from the present finding that autophagy ability and phagocytosis of introduced Aβ-FITC is decreased in UMSCs after long-time culture. In addition, LAMP1, which protects the lysosomal membrane from degradation, is dispersed in late passaged UMSCs. Moreover, LysoTracker Red labeled lysosomes confirmed the phenomena of dispersed distribution in the cytoplasm of late passaged UMSC, indicating swelling lysosomes caused by lysosomal membrane permeabilization [65], resulting in massive lysosomal leaking in cytosolic acidity, uncontrolled breakdown of cell components and cell death by necrosis [65]. Therefore, we believe that the lysosomal dysfunction in UMSC after long-time culture causes cellular waste accumulation and cellular component recycling obstacles, eventually affecting cell homeostasis and cell activity.

## Conclusions

The present study provides the first evidence that UMSCs, after long-time culture, can cause mitochondrial and lysosome dysfunction. It results in energy metabolism decline, cellular waste disposal and recycling obstacles, and entry into senescence that eventually reduce cell activity. Therefore, the morphology and function of mitochondria and lysosomes can be regarded as the two important parameters for assessing stem cell viability and therapeutic potentiality. In addition, they can also serve as two important aspects for optimizing the culture condition of MSCs.

## Supplementary Information

**Additional file 1. Figure S1** Flow cytometer analysis of UMSC-P4 and UMSC-P9 with cell surface markers.

**Additional file 2. Figure S2.** Fluorescence images of ROS and flow cytometry of apoptotic cells of, UMSC-P4 and UMSC-P9 treated with/ without H2O2 treatment. **a** Flow cytometry of apoptotic cells in UMSC-P4 and UMSC-P9 using PI/Annexin V staining. **b** Representative fluorescence images of ROS levels detected by oxidantsensing probe H2-DCFDA in UMSC-P4 and UMSC-P9. **c** Phase contrast images of UMSC-P4 and UMSC-P9 stained by Giemsa after treated without or with 50 μm H_2_O_2_ for 24 hours. Scale bar, 50 μm. **d** Flow cytometry of apoptotic cells in UMSC-P4 and UMSC-P9 treated with 50mm H_2_O_2_ for 24 hours using PI/Annexin-V staining. **e** Fluorescence images of ROS levels detected by H2-DCFDA in UMSC-P4 and UMSC-P9 treated with 50 μm H_2_O_2_ for 24 hours.

**Additional file 3. Figure S3.** Relative significant expression level changes of intermediate metabolites of glycolysis pathway (a) and TCA (b) in the supernatant of UMSC-P4 and UMSC-P9.

**Additional file 4. Table S1.** List of relative expression levels of identified metabolites related energy metabolism in the supernatant of UMSC-P4 and UMSC-P9. Related to Figure 5A, 5B and Figure S3.

**Additional file 5. Table S2.** List of expression levels of identified metabolites related energy metabolism in the cell lysate of UMSC-P4 and UMSC-P9. Related to Figure 5F.

**Additional file 6. Table S3.** List of transcripts of 120210 genes and their FPKM from transcriptome sequence in UMSC-P4 and UMSC-P9. Related to Figure 7A and 7B.

**Additional file 7. Table S4**. List of transcripts upregulated from transcriptome sequence in UMC-P9 versus UMSC-P4. Related to Figure 7 and Figure 8.

**Additional file 8. Table S5.** List of transcripts downregulated from transcriptome sequence in UMSC-P9 versus UMSC-P4. Related to Figure 7 and Figure 8.

## Data Availability

All related data and materials are available under request.

## Abbreviations

MSCs: Mesenchymal Stem cells
UMSCs: Human umbilical cord MSCs
P4: Passage 4
P9: Passage 9
I/R: Ischemia/reperfusion
PBMCs: Peripheral blood mononuclear cells
CFSE: Carboxyfluorescein succinimidyl ester
TEM: Transmission electron microscope
TMRM: Tetramethylrhodamine methyl ester
ROS: Reactive oxygen species
GPX4: Glutathione peroxidase 4
HK1: Hexokinase 1
HK2: Hexokinase 2
PFK: Phosphofructokinase
LDHA: Lactate dehydrogenase A
CS: Citrate synthase
ACO2: Aconitase 2
FH: Fumarase
SDHA: Succinate dehydrogenase A
TCA: Tricarboxylic acid
OXPHOS: Oxidative phosphorylation
HPLC/MS: High-performance liquid chromatography/mass spectrometry
GC/MS: Gas chromatography/MS
LAMP1: Membrane glycoprotein 1
GO: Gene ontology
Aβ-FITC: FITC-beta amyloid
