# Supplementary figures and images for: Biological activity reduction and mitochondrial and lysosomal dysfunction of mesenchymal stem cells aging in vitro
Source: Stem Cell Res Ther. 2022 Aug 13;13:411. doi: 10.1186/s13287-022-03107-4 (PMC9375398; doi:10.1186/s13287-022-03107-4)

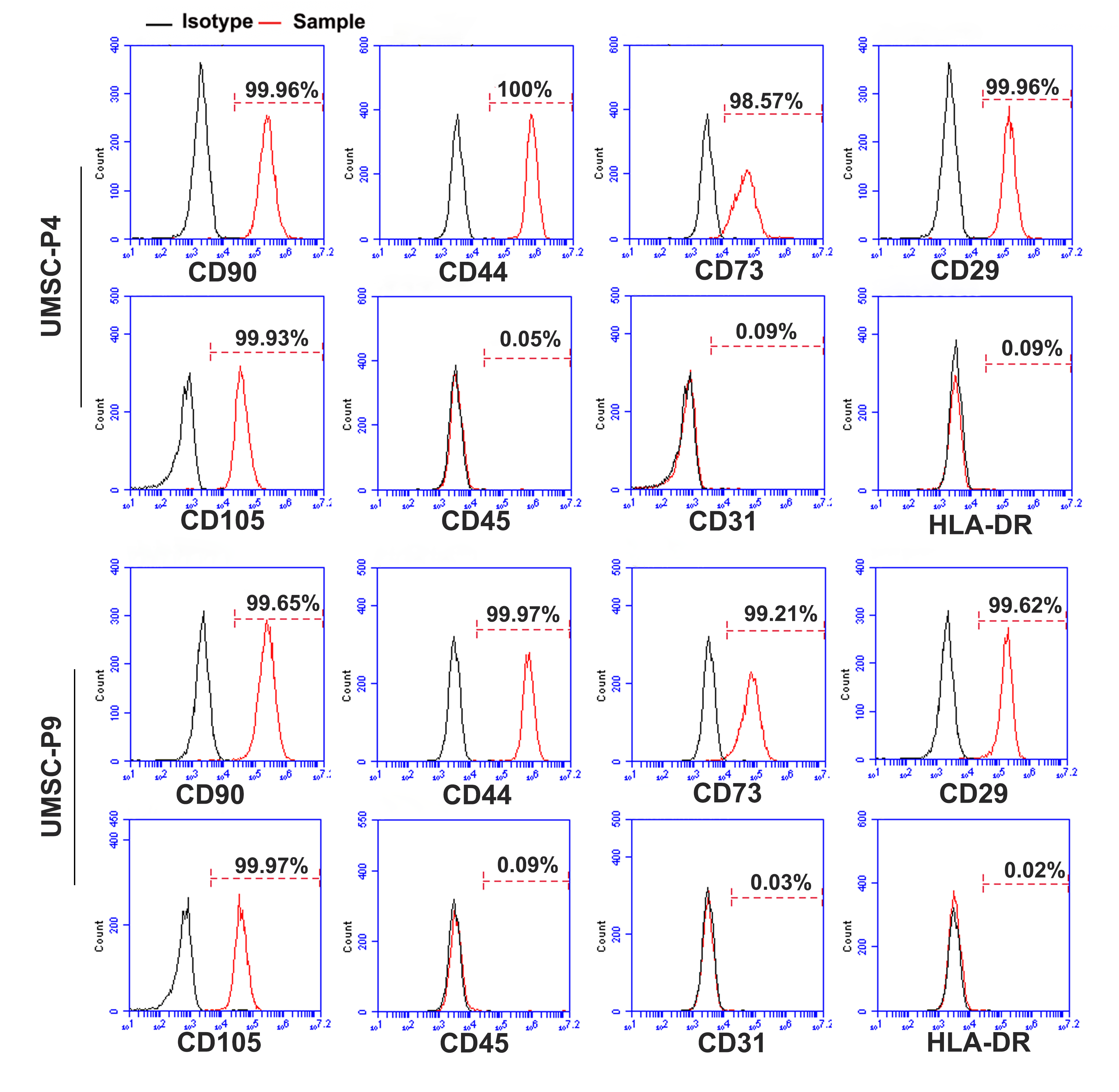

Supplement: Supplementary file 1 — Additional file 1. Figure S1 Flow cytometer analysis of UMSC-P4 and UMSC-P9 with cell surface markers. [file 13287_2022_3107_MOESM1_ESM.tif]

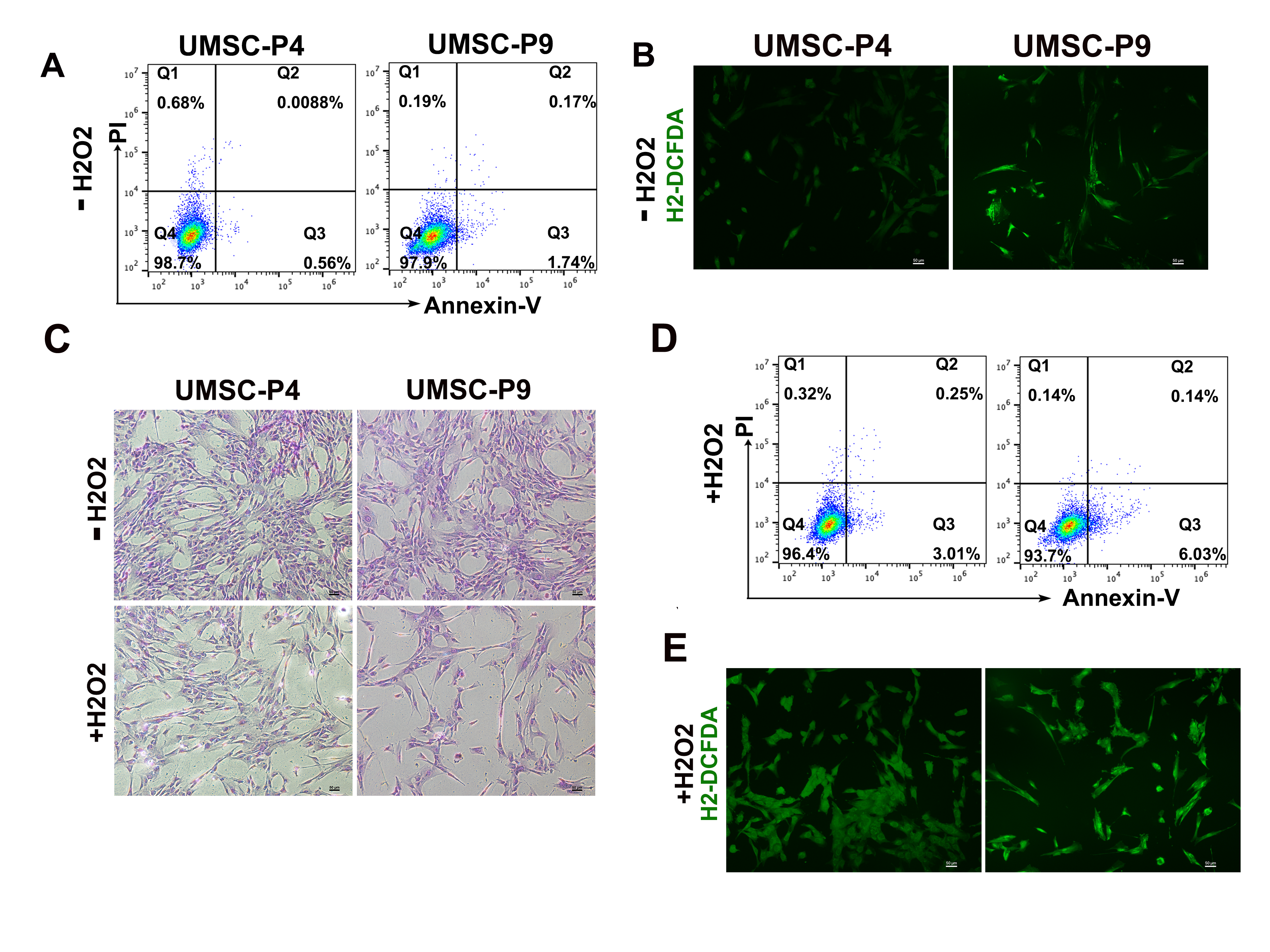

Supplement: Supplementary file 2 — Additional file 2. Figure S2. Fluorescence images of ROS and flow cytometry of apoptotic cells of, UMSC-P4 and UMSC-P9 treated with/ without H2O2 treatment. a Flow cytometry of apoptotic cells in UMSC-P4 and UMSC-P9 using PI/Annexin V staining. b Representative fluorescence images of ROS levels detected by oxidantsensing probe H2-DCFDA in UMSC-P4 and UMSC-P9. c Phase contrast images of UMSC-P4 and UMSC-P9 stained by Giemsa after treated without or with 50 μm H2O2 for 24 hours. Scale bar, 50 μm. d Flow cytometry of apoptotic cells in UMSC-P4 and UMSC-P9 treated with 50mm H2O2 for 24 hours using PI/Annexin-V staining. e Fluorescence images of ROS levels detected by H2-DCFDA in UMSC-P4 and UMSC-P9 treated with 50 μm H2O2 for 24 hours. [file 13287_2022_3107_MOESM2_ESM.tif]

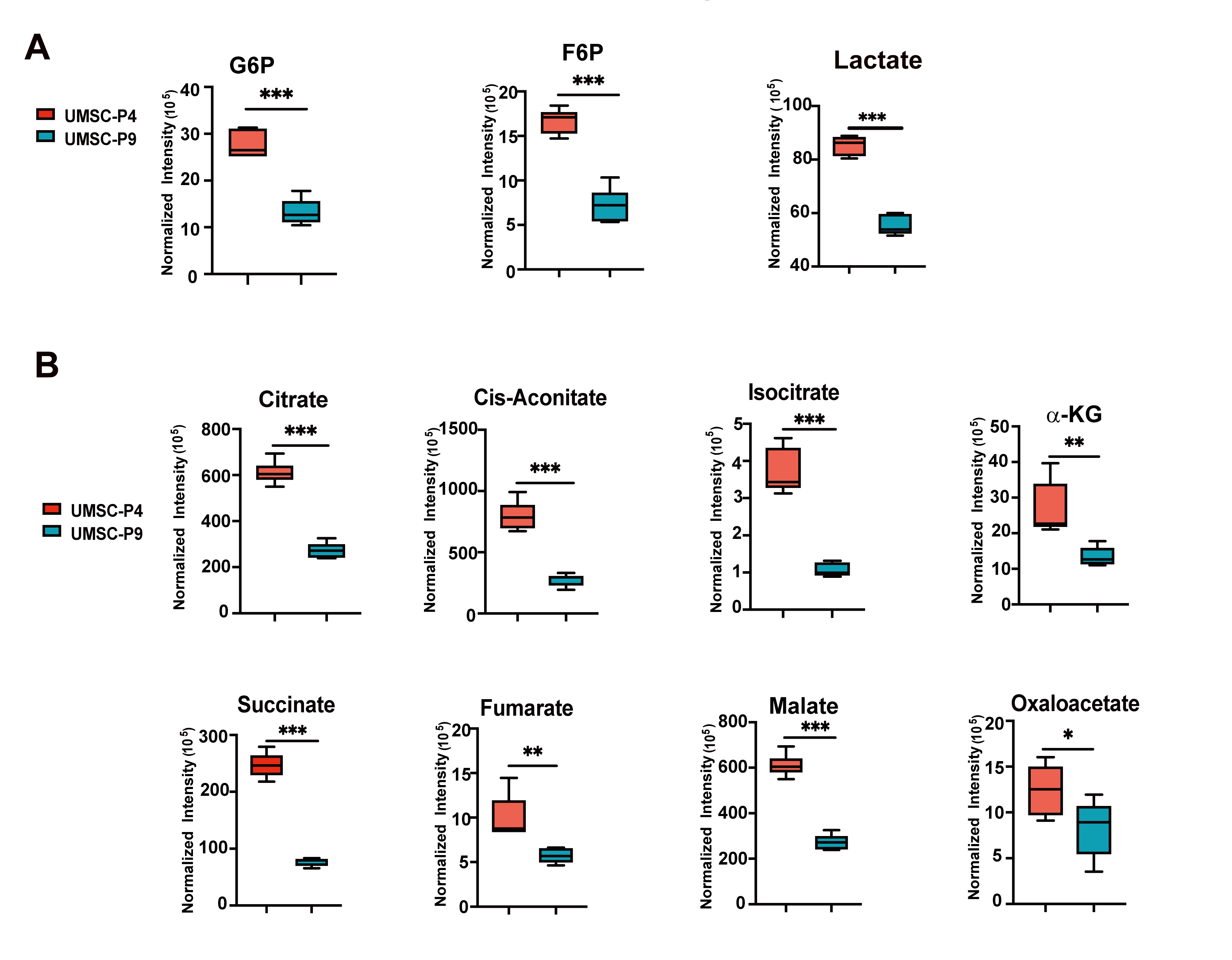

Supplement: Supplementary file 3 — Additional file 3. Figure S3. Relative significant expression level changes of intermediate metabolites of glycolysis pathway (a) and TCA (b) in the supernatant of UMSC-P4 and UMSC-P9. [file 13287_2022_3107_MOESM3_ESM.tif]
